# Study of the Mechanical and Fracture Properties of Lightweight Concrete with Various Combinations of Polypropylene Fibers

**DOI:** 10.3390/ma19030611

**Published:** 2026-02-04

**Authors:** Kristýna Hrabová, Jaromír Láník, Petr Lehner

**Affiliations:** 1 Faculty of Civil Engineering, Institute of Building Testing, Brno University of Technology, Veveří 331/95, 602 00 Brno, Czech Republic; kristyna.hrabova@vutbr.cz (K.H.);; 2 Department of Structural Mechanics, Faculty of Civil Engineering, VSB—Technical University of Ostrava, L. Podéště 1875, 708 00 Ostrava-Poruba, Czech Republic

**Keywords:** polypropylene fibers, lightweight concrete, mechanical parameters, fracture parameters

## Abstract

This article examines how hybrid polypropylene fibers of three different lengths affect the mechanical and fracture properties of lightweight structural concrete with lightweight ceramic aggregate. Four mixtures were produced: a reference lightweight concrete and three fiber-reinforced variants with total dosages of 3, 6, and 9 kg/m^3^ in a fixed length ratio of 4:1:1. Standard tests determined the bulk density, cube compressive strength, splitting tensile strength, modulus of elasticity, and fracture parameters using a three-point bend test. Compared to the reference concrete, the fibers did not significantly change the compressive strength but consistently increased the tensile strength and energy absorption after cracking. The highest fracture energy and toughness were obtained at the highest dosage, while excessive fiber content reduced the static compressive modulus.

## 1. Introduction

With the growing emphasis on reducing carbon dioxide emissions and extending the life cycle of buildings, the long-term durability of concrete structures is becoming a key requirement. Efforts are, therefore, being made to minimize the number and extent of cracks, reduce water and chemical permeability, and strengthen corrosion protection [1,2]. Steel reinforcements are susceptible to corrosion, so reinforced concrete elements can degrade if they are poorly designed or constructed. In addition, the production of steel meshes, and fibers represents a significant burden in terms of carbon footprint [3]. In this context, increasing attention is, therefore, being paid to lightweight concrete reinforced with polypropylene (PP) fibers. PP fibers are not subject to corrosion, which significantly reduces the risk of structural degradation and eliminates the need for steel reinforcement in certain types of applications. Thanks to their low density, they contribute to the overall lightening of the structure, while their presence in the concrete matrix reduces the formation of microcracks, improves cohesion, and increases resistance to water and chemical penetration [4,5]. Lightweight concrete with PP fibers is therefore a promising material that can help both extend the life of buildings and further reduce the carbon footprint of the construction sector [6,7].

Fiber-reinforced concrete (FRC) is a proven way to mitigate the brittle fracture behavior of cement composites. Current summary works confirm that fibers improve flexural tensile strength, fracture energy, and ductility, while the effect on compressive strength is limited [5,8]. At the same time, it is necessary to control the workability of the fresh mixture with an appropriate dose of superplasticizer [9]. In recent years, the use of PP fibers (≈38–54 mm) has become particularly popular, as they significantly increase residual flexural strength and fracture energy even in high-strength matrices, including UHPC. In such cases, the key is to design the appropriate dosage and dispersion of fibers in the matrix [10,11]

For lightweight concretes with ceramic aggregates, such as Liapor (Liapor, s.r.o., Vintirov, Czech Republic), recent studies show that the combination of microsilica and PP fibers leads to a sharp increase in toughness and residual strength [12,13]. In addition to static effects, benefits under dynamic loading have also been documented—PP fibers increase energy dissipation and peak stress at higher deformation rates [14,15].

In terms of standards, Eurocodes are undergoing extensive updates. The second generation of EN 1992 (Eurocode 2) [16] is gradually being finalized with the aim of easier usability and harmonization, as the new parts will be distributed to national standardization bodies by March 2026, with publication scheduled for 2027 [17]. Therefore, there is great potential for research and the contribution of new knowledge in this field.

Based on these findings, this study is motivated by the need to deepen current knowledge and address several gaps that remain insufficiently explored. Although the positive influence of PP fibers on toughness, residual strength, and fracture energy has been repeatedly demonstrated, most existing studies focus on either conventional or high-performance concretes, while lightweight concretes with ceramic aggregates are much less extensively investigated. Furthermore, research rarely compares different combinations, lengths, or dosages of PP fibers in the same lightweight matrix, even though fiber morphology and synergies between different fiber types can play a decisive role in achieving optimal mechanical behavior [18,19,20].

The main objectives were to experimentally evaluate how different combinations and dosages of PP fibers affect the mechanical properties (compressive, tensile, and flexural strength) of lightweight concrete. Furthermore, to identify potential synergies between different fiber types or lengths and to determine the optimal fiber configurations for lightweight matrices with ceramic aggregates such as Liapor. This will contribute to data that can support the refinement of design models and the development of future standard provisions for fiber-reinforced lightweight concretes. This study should be considered innovative in the field of fiber-reinforced concrete (FRC) for several reasons. Unlike most existing research that focuses on conventional or high-performance concretes, this work systematically investigates lightweight structural concrete with ceramic aggregate (Liapor) combined with hybrid polypropylene fibers of three different lengths. The experimental program not only evaluates compressive, tensile, and elastic properties but also provides a comprehensive analysis of fracture mechanics parameters, including effective fracture toughness and specific fracture energy, using the standardized RILEM FMC 1 method.

## 2. Materials and Methods

**Figure 1:**
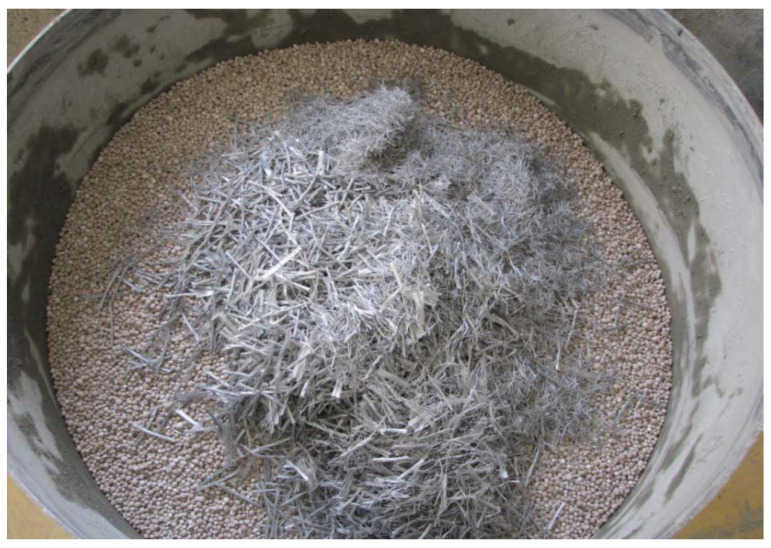
Demonstration of concrete mix preparation with Liapor aggregate and PP fibers.

The purpose of the study is to assess how the basic properties of lightweight Liapor concrete differ when the same recipe is used, but with the addition of PP fibers of different lengths and in different proportions. PP fibers with lengths of 54, 38, and 19 mm were selected as the dispersion reinforcement. Highly effective Forta Ferro fibers [10,21], which combine monofilament PP fibers with fibrous “mesh” fibrillated tapes, were used to produce test samples (see Figure 1). This mixture ensures the reduction in shrinkage deformations in the early and hardened stages of concrete, increases its impact resistance, improves its behavior under cyclic stress, and significantly supports the overall toughness of the material. In addition, the fibers are designed to withstand high operating loads over the long term. The detailed procedure for preparing the mixtures, their composition, and a description of the test methods used are given in the following section.

### 2.1. Concrete Mixtures and Samples

In cement composites where PP fibers are used as dispersed reinforcement, it is very important to pay attention to the correct production technology and the pitfalls in production, processing of fresh concrete and placement of test samples. To produce 1 m^3^ of fresh concrete of each of the four mixtures, the proportions and weight of individual parts were determined according to Table 1. The mixture is marked with C for concrete, L for lightweight, and the weight of fibers of three lengths in the order of 54 mm, 38 mm, and 19 mm. The ratio of PP fibers was selected to exploit the complementary roles of macro- and microfibers in the concrete matrix. The longest fibers (54 mm) dominate the mixture to ensure effective post-peak load transfer and crack bridging, while shorter fibers (38 mm and 19 mm) are included in smaller proportions to control early microcracks and plastic shrinkage.

The procedure for producing fresh concrete depended on the type of filler used. Therefore, the fibers were dosed into the mixer with upper blades in a weight ratio to the aggregate. Pre-soaking water was applied to the already dispersed fibers. The material was therefore added in the following order: Liapor, PP fibers, pre-soaking water, fine quarried aggregate, cement, fly ash and mixing water.

To determine the physical and mechanical properties of concrete, test samples were made in the shape of cubes with an edge length of 150 mm and beams with dimensions of 100 × 100 × 400 mm. For each set, 9 beams and 6 cubes were produced, which were demolded after 24 h and stored in a humid environment with 90% relative humidity at a temperature of *t* = 20°. The quality of the concrete surface separated from the mold was ensured by using separation or formwork removal agents. Therefore, no damage to the test samples occurred during demolding. The removed samples were exposed to uniform conditions during curing, where moist storage ensured sufficient hydration of the cement and thus prevented shrinkage and the formation of cracks or deformation of the test samples. The samples were placed in a container with sufficient water and covered with foil, thus fulfilling the conditions for protecting the concrete surface and achieving the highest possible strength of the cement composites.

### 2.2. Testing Program

The experimental program was designed to comprehensively cover the key mechanical and fracture characteristics of fiber-reinforced concrete, both through standardized tests and methods specifically suitable for composites with dispersed reinforcement. This approach ensured that the results were sufficiently informative and allowed for a comparison of individual mixtures with different combinations of PP fibers in the context of lightweight Liapor-based concrete.

The program first included a test of bulk density, which is a basic material parameter affecting the strength and deformation properties of concrete. After demolding, all samples were carefully measured, weighed, and the results were evaluated according to the EN 12390-7 [22] methodology, with three separately prepared samples tested for each mixture.

This was followed by a compressive strength test, which was performed on cubic bodies with an edge length of 150 mm, prepared and treated in accordance with EN 12390-3 [23]. Each sample was continuously loaded at a rate of 0.5 MPa/s until destruction, which made it possible to determine the compressive strength based on the maximum force achieved and the known area of the loaded wall. Again, three samples from each concrete mixture were tested.

The next step was to test the splitting tensile strength. This test was carried out in accordance with EN 12390-6 [24], again on 150 mm cubes. The samples were loaded at a rate of 0.05 MPa/s until failure, and the resulting strength was calculated from the maximum force achieved, the contact area, and the relevant dimensions of the body. This test is particularly important for fiber-reinforced concrete, as the presence of fibers can significantly affect the course and propagation of cracks.

**Figure 2:**
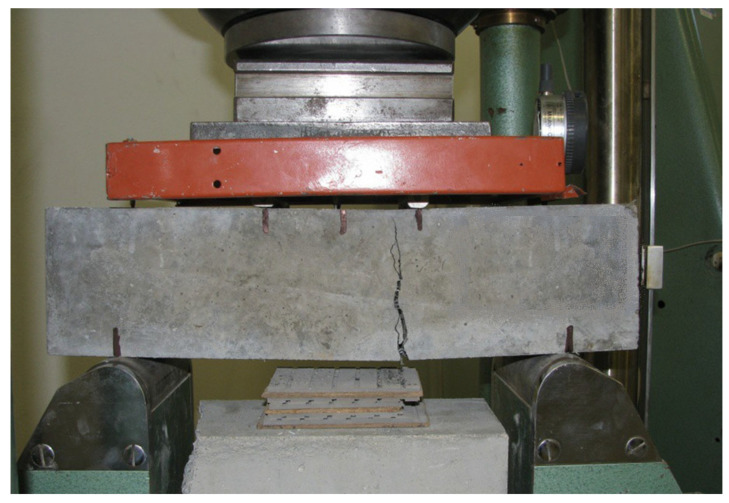
Four-point bending setup.

Subsequently, the static modulus of elasticity was determined in accordance with EN 12390-13 [25], which is a parameter determining the stiffness of the material and its ability to undergo elastic deformation. The test was performed on prismatic samples measuring 100 × 100 × 400 mm (see Figure 2), which were gradually loaded in several steps and then unloaded. The elastic deflections measured were used to calculate the modulus of elasticity using the appropriate formula. The values obtained are essential, for example, for numerical modeling or the assessment of structural element deformations.

The most difficult and at the same time most important part of the entire program was the determination of fracture parameters, because fracture mechanics provides information about the behavior of the material in the vicinity of cracks, its ability to resist their propagation, and the energy conditions during fracture. The three-point bending test was performed on prismatic samples of size 100 × 100 × 400 mm. The entire test was performed in accordance with RILEM FMC 1 [26]. In order to evaluate controlled crack propagation, all samples were pre-notched with a diamond wheel to a depth of one-third of the cross-section height. The samples were placed in a Hecker FPZ 100/10 press with a support span of 300 mm and loaded at a constant deformation rate of 0.05 mm/min. Both the loading force and vertical deflection were continuously recorded during the test. Five beams were prepared and tested for each mixture and after that detailed analysed in the StiCrack program. This made it possible to determine several key fracture parameters, such as the static modulus of elasticity, effective fracture toughness and specific fracture energy *GF*, which characterize the energy required to create and propagate a crack. These values are key to describing the behavior of fiber-reinforced concrete under overload, for the design of structural elements, and for the validation of numerical models. The entire experimental procedure thus made it possible to comprehensively assess the properties of lightweight Liapor concrete with various combinations of PP fibers and to create a comprehensive set of data that can be used both in science and in practice when applying fiber-reinforced concrete in structures.

Information about all tests from the program is clearly presented in Table 2.

## 3. Results

This chapter presents the results according to the test program described above. The measurements determined the average values of individual variables and the standard deviation. In addition, at the end of the chapter, there is a summary table showing the percentage differences for individual tests and mixtures.

### 3.1. Bulk Density

**Figure 3:**
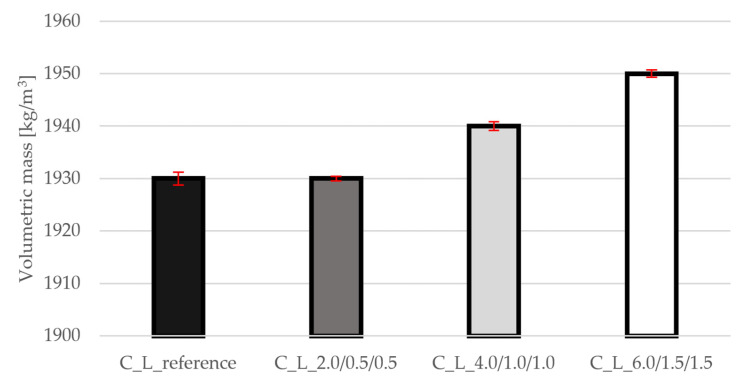
Results of bulk density for all mixtures.

The bulk density results are shown in Figure 3. The values are within ±1% compared to the reference mixture, which confirms that the lightweight ceramic Liapor aggregate remains the dominant factor determining the density. This result is important for the design of structures, since maintaining low weight is a key parameter of lightweight concretes. The graph also shows very small standard deviations that do not deviate outside the range.

### 3.2. Compressive Strength

**Figure 4:**
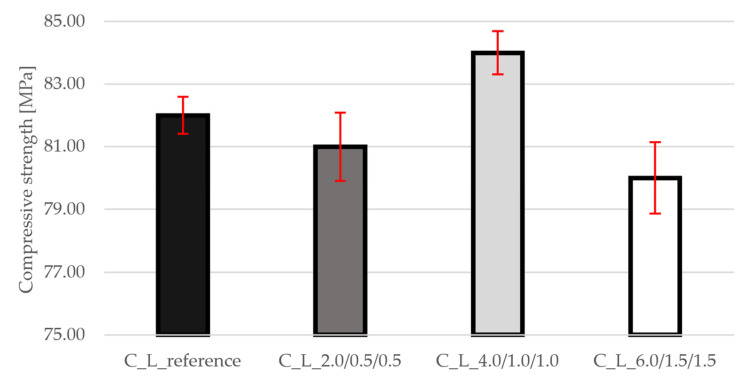
Results of compressive strength for all mixtures.

Figure 4 shows the results of compressive strength. All mixtures achieve comparable values (approx. 80–84 MPa), with differences not exceeding 2. Slight deviations can be attributed to differences in the workability of mixtures at higher fiber content. The graph shows small standard deviations that do not deviate outside the range.

### 3.3. Tensile Strength

**Figure 5:**
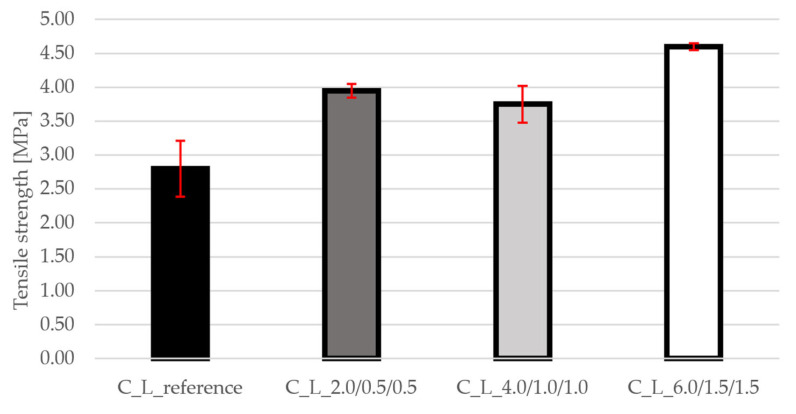
Results of tensile strength for all mixtures.

The results of the tensile strength (Figure 5) show a significant influence of fibers. The increase compared to the reference mixture is 41% for the C_L_2.0/0.5/0.5 mixture, 34% for the C_L_4.0/1.0/1.0 mixture and up to 64% for the C_L_6.0/1.5/1.5 mixture. This trend confirms the effective transmission of tensile stresses through the fibers and the limitation of microcrack propagation. The highest increase was recorded for the mixture with the highest fiber content, indicating synergy between macro- and microfibers. The graph shows small standard deviations, indicating that the greatest dispersion was in the reference concrete, but conversely decreased to a very small value in the fibre concrete.

### 3.4. Elastic Modulus

**Figure 6:**
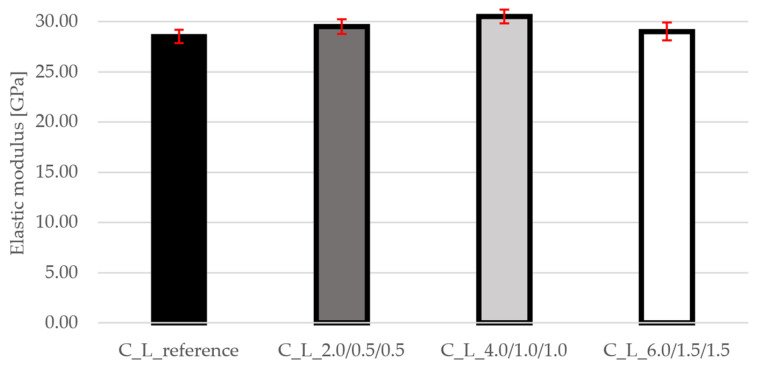
Results of elastic modulus for all mixtures.

The modulus of elasticity (Figure 6) shows higher values at higher fiber content. While the C_L_4.0/1.0/1.0 mixture achieves the highest stiffness, the C_L_6.0/1.5/1.5 mixture shows a decrease to approximately 29 GPa. The C_L_4.0/1.0/1.0 mixture appears to have an optimal fiber combination that improves cohesion and reduces microcracking, leading to higher stiffness. At this point, the fiber content is sufficient for effective stress transfer without significantly disrupting the matrix. At the highest fiber content, poorer processability of the mixture and local voids may occur. These factors increase heterogeneity and reduce the effective modulus of elasticity. In addition, a higher fiber content may result in more fiber–matrix interfaces, leading to slightly higher deformability. These are precisely the reasons why the C_L_4.0/1.0/1.0 mixture shows the best results. The graph also shows very small standard deviations that do not deviate outside the range.

### 3.5. Effective Fracture Toughness

**Figure 7:**
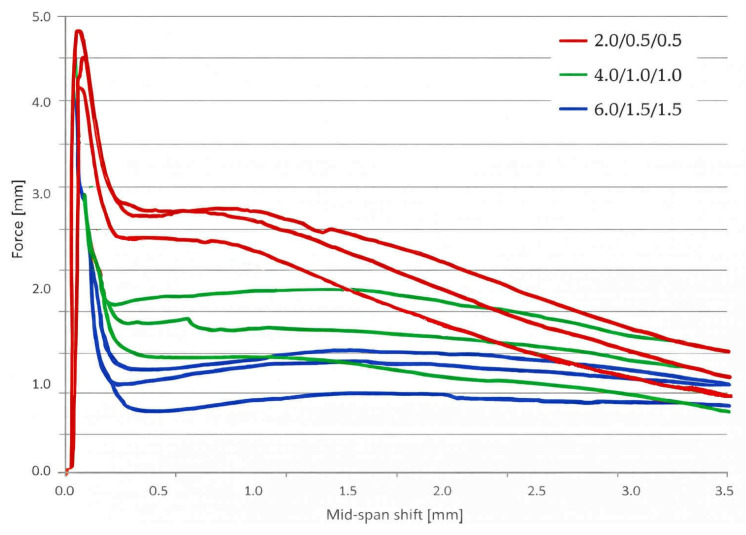
Recorded force–displacement diagram for all samples of all mixtures.

As mentioned above, records from force–displacement diagrams were used to calculate the fracture parameters. These are shown in Figure 7 for all mixtures.

**Figure 8:**
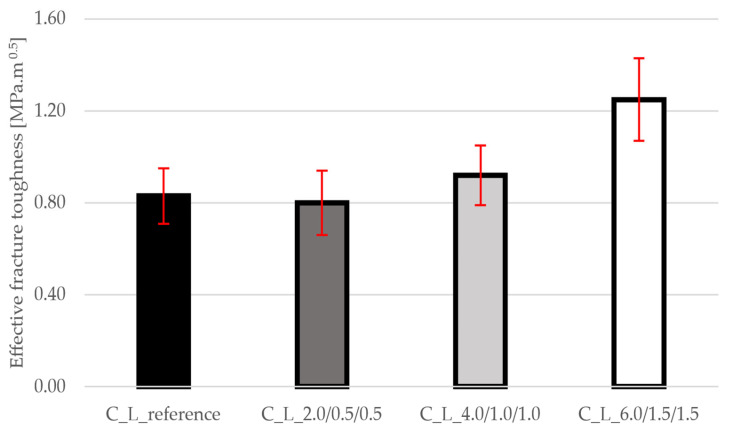
Results of effective fracture toughness for all mixtures.

Figure 8 shows a significant increase in effective fracture toughness up to the last mixture. While the mixture with low fiber content shows a slight deterioration or improvement, the C_L_6.0/1.5/1.5 mixture achieves up to +51% compared to the reference mixture. This parameter is key to crack propagation resistance and confirms the positive effect of fiber on the fracture behavior of concrete. The standard deviations shown in the graph are the same for all mixtures and do not deviate from the range.

### 3.6. Specific Fracture Energy

**Figure 9:**
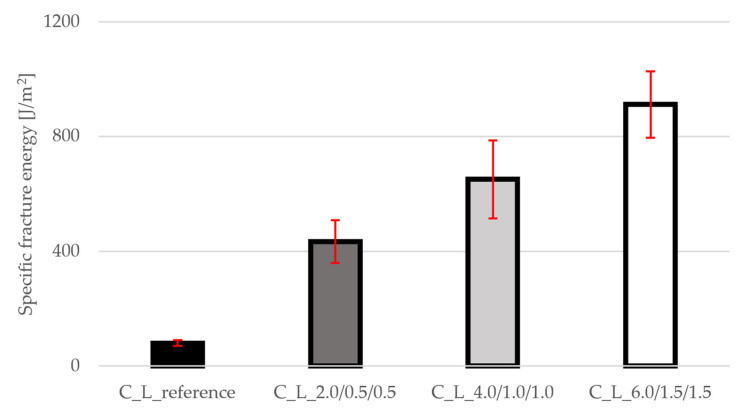
Results of Specific fracture energy for all mixtures.

The specific fracture energy (Figure 9) shows the most significant changes. The value increased from 80 J/m^2^ for the reference mixture to 434 J/m^2^ (C_L_2.0/0.5/0.5), 651 J/m^2^ (C_L_4.0/1.0/1.0) and up to 911 J/m^2^ for the C_L_6.0/1.5/1.5 mixture, representing an increase of more than 1000%. This result demonstrates the ability of fibers to absorb energy after cracking and significantly improves the ductility of the material. In practice, this means greater structural safety under overload and better resistance to sudden failure. The standard deviations showed that the reference mixture has minimal variability, but the other mixtures do not deviate from the range.

### 3.7. Comparison of Results

A summary of the data as a percentage difference from the reference mixture is given in Table 3. The results show that the addition of PP fibers has virtually no effect on the bulk density of lightweight concrete with Liapor (C_L_2.0/0.5/0.5: 0%, C_L_4.0/1.0/1.0: +1%, C_L_6.0/1.5/1.5: +1%), while the compressive strength remains within the same class with minimal deviations (−1%, +2%, −2%), confirming that the fibers primarily affect tensile and fracture behavior; tensile strength in splitting increases significantly even at the lowest dosage (+41%) and peaks at the highest (+64%) thanks to effective fiber bridges that limit the initiation and development of microcracks. The static modulus of elasticity increases slightly in relative terms (+4%, +7%, +2%), with the highest stiffness being exhibited by the medium dose, which indicates optimal dispersion and orientation of the fibers for stress transfer in the elastic range; at the highest dose, the increase in modulus is limited, probably due to the greater heterogeneity of the matrix.

The fracture parameters reflect the significant contribution of the fibers: the effective fracture toughness decreases slightly at low dosage (−4%) but increases at medium dosage (+11%) and shows a significant improvement at the highest dosage (+51%), reflecting effective bridging, wedging, and pull-out of longer fibers supplemented by shorter types. The most dramatic changes are brought about by the specific fracture energy, which increases by +442%, +712%, and +1038% compared to the reference, demonstrating the material’s significantly higher ability to dissipate energy after crack initiation and the transition from brittle to more ductile post-peak behavior. in practical terms, this means better crack control, higher safety under overload, and the option of choosing a medium dose for maximum stiffness or the highest dose for maximum tensile response, toughness, and energy dissipation without increasing the weight of the elements.

## 4. Discussion

The results demonstrate that hybrid PP fibers substantially modify the tensile and fracture response of lightweight Liapor concrete while leaving bulk density and compressive strength essentially unchanged. The practically identical bulk density across all fiber dosages (0–1% relative change) confirms that the low-density PP fibers, added in small volumetric fractions, do not alter the self-weight of the material; density remains governed by the lightweight ceramic aggregate and the cementitious matrix. This is beneficial for structural applications where reducing dead load is a primary motivation for using lightweight concrete.

In compression, all mixtures remained within the same strength class as the reference, with relative deviations of −1%, +2%, and −2% for C_L_2.0/0.5/0.5, C_L_4.0/1.0/1.0, and C_L_6.0/1.5/1.5, respectively. This behavior is consistent with the established understanding that synthetic fibers have a limited ability to raise peak compressive strength in cementitious composites [7,9]. Minor differences likely reflect changes in fresh-mix rheology and local heterogeneity at higher fiber volumes (e.g., increased number of fiber–matrix interfaces and entrapped voids), rather than a fundamental modification of the compressive failure mechanism. Put differently, the fibers do not strengthen the load-bearing skeleton in compression; their key role emerges once cracking initiates.

The splitting tensile strength exhibits the most immediate mechanical benefit, increasing by +41%, +34%, and +64% for the three fiber dosages. The improvement is attributed to crack-bridging and stress transfer through fiber pull-out and anchorage, which delay crack initiation and reduce stress concentrations at the aggregate–matrix interfaces. The non-monotonic ordering between the low and medium dosages suggests that tensile performance depends not only on fiber volume, but also on dispersion and orientation achieved during mixing and casting—factors that are known to influence the efficiency of load transfer across developing cracks in fiber-reinforced concretes [9,11]. The highest dosage appears to provide a sufficient density of effective bridges across multiple crack scales, delivering the largest gain.

The static modulus of elasticity shows a mild increase relative to the reference, with the maximum at the medium dosage. This pattern indicates that a moderate amount of fibers can slightly enhance the effective stiffness in the nominally elastic regime, likely by stabilizing microcrack closure at low strains and improving the integrity of the interfacial transition zones. At the highest dosage, the net gain in modulus is smaller, which can be rationalized by the counteracting effects of increased heterogeneity, fiber clustering risk, and local compliant interfaces. Importantly, given the inherently lower modulus of lightweight aggregates compared to normal-weight counterparts, large stiffness gains from PP fibers should not be expected.

Fracture parameters capture the most consequential impact of hybrid PP reinforcement. Effective fracture toughness rises with dosage, while the specific fracture energy increases dramatically. The slight decrease in toughness at the lowest dosage likely reflects an insufficient population of effectively oriented bridges in the notch ligament and normal test-to-test scatter inherent to three-point bending with prenotches.

The medium dosage (C_L_4.0/1.0/1.0) delivers the highest relative gain in modulus while already providing substantial growth in fracture energy, suggesting a favorable balance between stiffness retention and ductility. The highest dosage (C_L_6.0/1.5/1.5) maximizes tensile strength, toughness, and energy, but—characteristically for high fiber volumes—offers only a limited additional benefit for elastic stiffness and may be more sensitive to workability and compaction.

Finally, two practical considerations deserve emphasis. First, the negligible density change confirms that the mechanical upgrades come without a penalty in self-weight—a key benefit for lightweight structural elements. Second, the sensitivity of fracture metrics to fiber dosage underscores the importance of production technology: dosing sequence, pre-wetting strategy for fibers, mixing energy, and compaction directly condition dispersion and orientation, and thus the realized gains. Future work should focus, among other things, on the durability parameters of these concrete mixtures [27,28]. Such data would facilitate refined constitutive modeling and support the forthcoming updates to design provisions for fiber-reinforced lightweight concretes [17]. All of the above results naturally have an impact on possible numerical or analytical models. In general, for cement composites, fracture work and fracture energy increase with the amount of dispersed reinforcement material used. The higher the mass fraction of PP fibers, the more work is required to achieve the greater energy needed to create a crack and cause complete failure of the beam. This is directly proportional to the calculation of flexural strength, which is the main criterion in numerical analysis.

## 5. Conclusions

The following conclusions were drawn from the above results and discussions:

(1) The addition of PP fibers significantly increases the tensile strength and fracture energy of lightweight concrete, with the highest increase observed in the C_L_6.0/1.5/1.5 mixture, where the splitting tensile strength rose by +64% (from approx. 4.8 MPa to 7.9 MPa) and the specific fracture energy increased by +1038% (from 80 J/m^2^ to 911 J/m^2^).
(2) Cubic compressive strength remains virtually unchanged for all mixtures, with values around 80–84 MPa and deviations within ±2%, so all mixtures can be classified in the same strength class.
(3) The combination of three fiber lengths is highly effective, as macrofibers ensure post-peak load transfer and microfibers contribute to crack control. For example, the mixture C_L_4.0/1.0/1.0 achieved the highest static modulus of elasticity (≈31 GPa, +7% compared to reference), while still providing a substantial increase in fracture energy (651 J/m^2^, +712%).

## Figures and Tables

**Table 1:** Composition of concrete mixtures.

	C_L_Reference	C_L_2.0/0.5/0.5	C_L_4.0/1.0/1.0	C_L_6.0/1.5/1.5
Portland Cement CEM I 42.5R [kg]	440	440	440	440
Fly ash [kg]	80	80	80	80
Fine aggregate 0/4 [kg]	700	700	700	700
Coarse aggregate—Liapor600 4/8 [L]	1	1	1	1
Superplasticizer (Sika ViscoCrete 1035) [kg]	5	6	6	6
Water [L]	191	193	193	199
W/C ratio	0.43	0.44	0.44	0.45
Forta Ferro 54 mm [kg]	0.0	2.0	4.0	6.0
Forta Ferro 38 mm [kg]	0.0	0.5	1.0	1.5
Forta Ferro 19 mm [kg]	0.0	0.5	1.0	1.5

**Table 2:** List of tests and standards used.

Name	Standard	Size of Sample	Number of Samples
Bulk density	EN 12390-7 [22]	150 × 150 × 150 mm	3 per mixture
Compressive strength test	EN 12390-3 [23]	150 × 150 × 150 mm	3 per mixture
Splitting tensile strength	EN 12390-6 [24]	150 × 150 × 150 mm	3 per mixture
Static modulus of elasticity	EN 12390-13 [25]	100 × 100 × 400 mm	3 per mixture
Three-point bending test (fracture toughness and energy)	RILEM FMC 1 [26]	100 × 100 × 400 mm	5 per mixture

**Table 3:** Relative difference in all test results to reference mixture.

Mixture	C_L_2.0/0.5/0.5	C_L_4.0/1.0/1.0	C_L_6.0/1.5/1.5
Bulk density	0%	1%	1%
Compressive strength	−1%	2%	−2%
Tensile strength	41%	34%	64%
Static modulus of elasticity	4%	7%	2%
Effective fracture toughness	−4%	11%	51%
Specific fracture energy	442%	712%	1038%

## Data Availability

The original contributions presented in this study are included in the article. Further inquiries can be directed to the corresponding author.
